# Identification and Evaluation of the Urinary Microbiota Associated With Bladder Cancer

**DOI:** 10.1002/cai2.70012

**Published:** 2025-05-25

**Authors:** Nannan Li, Lei Wang, Qin Yang, Fuqiang Li, Zhun Shi, Xiujie Feng, Liwei Zhang, Xiaojian Li, Xin Jin, Shida Zhu, Kui Wu, Ningchen Li

**Affiliations:** ^1^ College of Life Sciences University of Chinese Academy of Sciences Beijing China; ^2^ HIM‐BGI Omics Center, Hangzhou Institute of Medicine (HIM) Chinese Academy of Sciences, BGI Research Hangzhou China; ^3^ Guangdong Provincial Key Laboratory of Human Disease Genomics Shenzhen Key Laboratory of Genomics, BGI Research Shenzhen China; ^4^ BGI Genomics Harbin China; ^5^ BGI Shenzhen China; ^6^ Department of Urology Peking University Shougang Hospital Beijing China; ^7^ Peking University Wu‐Jieping Urology Center, Peking University Health Science Center Beijing China

**Keywords:** bladder cancer, microbiota, muscle‐invasive bladder cancer, non‐muscle‐invasive bladder cancer, urine

## Abstract

**Background:**

Bladder cancer is a common malignancy of the genitourinary system. Recent studies have confirmed the existence of microorganisms in urine. This study aimed to characterize changes in the urinary microbiota of Chinese bladder cancer patients and determine differences between patients with muscle‐invasive bladder cancer (MIBC) and those with non‐muscle‐invasive bladder cancer (NMIBC).

**Methods:**

Urine samples were collected from 64 patients with bladder cancer and 94 disease‐free controls using the clean catch method and sequenced by 16S rRNA gene sequencing. Sequencing reads were filtered by VSEARCH and clustered by UPARSE.

**Results:**

Significant associations were found between urinary microbiota and factors such as sex, age, and disease status. After age adjustment, differences in beta diversity were observed between healthy men and women, cancer patients and healthy controls, and NMIBC and MIBC patients. The cancer patients had an increased abundance of 14 bacterial genera, including *Stenotrophomonas*, *Propionibacterium*, and *Acinetobacter*. Notably, *Peptoniphilus* spp. were enriched in high‐risk MIBC patients, indicating their potential as a risk marker. Functional prediction via PICRUSt analysis suggested enriched metabolic pathways in specific disease groups. Furthermore, molecular ecological network analysis revealed differences based on sex and disease type.

**Conclusions:**

This significant microbial diversity indicates a potential correlation between urinary microbiota dysbiosis and bladder cancer, with implications for risk stratification and disease management. The identified urinary microbiota may serve as noninvasive markers for bladder cancer, warranting further validation in larger cohorts. This study provides a foundation for further research on the mechanisms of bladder cancer progression.

## Graphical abstract

Urinary microbiota in Chinese bladder cancer patients reveals distinct profiles between non‐muscle‐invasive and muscle‐invasive diseases, with potential biomarkers like *Peptoniphilus* spp. in high‐risk MIBC, suggesting a role in cancer progression. Dysbiosis may serve as a stratification marker for bladder cancer risk.

**Abbreviations**
Adonis: nonparametric multivariate analysis of variance
ANOSIM: analysis of similarity
BCM: men with bladder cancer
BCW: women with bladder cancer
FDR: false discovery rate
HM: healthy men
HW: healthy women
LDA: linear discriminant analysis
MIBC: muscle‐invasive bladder cancer
MRPP: multiresponse permutation procedure
NMIBC: non‐muscle‐invasive bladder cancer
OTU: operational taxonomic unit
PCoA: principal coordinate analysis
RMT: random matrix theory

## 1 Introduction

Bladder cancer is the tenth most common cancer worldwide [1]. The risk of bladder cancer increases with age, and the incidence in men is approximately four times greater than that in women [2]. According to the US National Comprehensive Cancer Network guidelines, bladder cancer is classified into muscle‐invasive bladder cancer (MIBC) and non‐muscle‐invasive bladder cancer (NMIBC) [3]. Approximately 75% of patients with bladder cancer are initially diagnosed with NMIBC, and the remaining 25% of patients suffer from MIBC [4]. Although the recurrence rate of NMIBC is high, it usually has a good prognosis. By contrast, MIBC is often associated with metastasis, and once it progresses, the prognosis is generally poor [5]. MIBC is a critical factor affecting the decision‐making process for bladder cancer treatment. Currently, the standard treatment for NMIBC patients is transurethral resection, whereas radical cystectomy is the criterion standard for MIBC patients. However, these methods are invasive and time‐consuming [6]. Treatment choice is crucial and significantly influences patient survival and recurrence rates. Therefore, early and accurate identification of NMIBC and MIBC is essential for tailoring treatment and improving patient survival [7].

Urine and the bladder were considered sterile for more than a century, but bacteria have now been found in urine through culture or 16S rDNA sequencing [8, 9]. In the past 5 years, research on the role of urinary microbiota in urogenital system diseases has developed rapidly. The urinary microbiota is influenced by various factors, such as age and sex [10, 11]. With increasing age, the urinary microbiota may change [10]. Tasha et al. found that the bacteria usually present in healthy female urinary microbiota were also found in the vagina, whereas the urinary microbiota in healthy male urine resembled that of the intestinal tract and skin [11]. Growing evidence suggests that urinary microbiota may be a key susceptibility factor in chronic urogenital diseases, including bladder cancer [12]. A potential interaction between urinary microflora and the development of urogenital cancer has been suggested. A typical example is the relationship between schistosomiasis infection in the bladder and an increased risk of bladder cancer in endemic regions, with chronic urinary schistosomiasis associated with the development of bladder squamous cell carcinoma [13].

The interaction between the microbiome and the host is complex. Bacteria can alter host signaling pathways to support cancer cell proliferation [14]. Some pathogenic microorganisms can damage host DNA through gene toxins, such as colicin produced by certain *Escherichia coli* strains, or indirectly by producing reactive oxygen species [15]. However, the role of urinary microbiota in the pathogenesis of bladder cancer has been less studied, and conclusive evidence linking it to bladder cancer is still lacking. The urinary microbiota may be crucial in maintaining urinary tract homeostasis [16]. One hypothesis suggests that the urinary microbiota may change the extracellular matrix, promoting or inhibiting bladder cancer progression [17]. Whether urinary microbiota influences the development of bladder cancer or whether bladder cancer alters the microbial composition, abundance, or diversity of the urinary microbiota requires further investigation.

Popović et al. reported that the genus *Fusobacterium* was more abundant in patients with bladder cancer, whereas *Viburnum*, *Streptococcus*, and *Corynebacterium* were more abundant in healthy controls [18]. Wu et al. found that specific bacterial genera, such as *Acinetobacter*, *Anaerococcus*, and *Rubrobacter*, were enriched in the urine of patients with bladder cancer, whereas genera such as *Serratia*, *Proteus*, and *Roseomonas* were less abundant [19]. Hussein et al. observed a relatively higher abundance of genera such as *Achromobacter*, *Actinomyces*, *Brucella*, and *Brevibacterium* in bladder cancer patients [20]. Other microorganisms that are more abundant in urine samples from patients with bladder cancer include *Escherichia–Shigella* [21], *Klebsiella* [22], and *Pseudomonas* [23]. Zoli et al. confirmed the consistency of bladder cancer‐associated microorganisms in both tissue and urine samples, identifying a common microbiome [24].

Few studies have focused on differences in urinary microbiota based on the stage of bladder cancer (MIBC vs. NMIBC), recurrence risk (high vs. nonrecurrent), and progression [25]. Sun et al. found that microbial diversity, specifically that of *Acinetobacter guillouiae* and *Brevibacillus agri*, in the tissues of NMIBC patients was greater than that in patients with MIBC. Conversely, *Ralstonia pickettii* and *R. mannitolilytica* are more abundant in patients with MIBC [26]. However, Hussein et al. found no significant differences in urinary microbial diversity (both alpha and beta diversity) between patients with NMIBC and those with MIBC [27]. Wu et al. reported that genera such as *Herbaspirillum*, *Porphyrobacter*, and *Bacteroides* were significantly enriched in cancer patients with a high risk of recurrence and progression [19]. Zeng et al. compared the microbial diversity of urine samples from recurrent and nonrecurrent bladder cancer patients, finding that patients with recurrent bladder cancer had a higher Shannon index, indicating greater diversity. However, no significant differences in microbial abundance were observed between the two groups [12].

Although research on human urine microbiota is increasing, findings on its role in bladder cancer are still inconsistent. Further investigations are needed to clarify the relationship between urinary microbiota and bladder cancer. Although some studies have explored microorganisms in bladder cancer tissues of NMIBC and MIBC patients [26], they have not been explored in urine. Therefore, we sought to investigate further differences in the urinary microbiota between patients with bladder cancer and healthy controls and between patients with NMIBC and those with MIBC to provide a more robust foundation for predicting this disease.

## 2 Methods

### 2.1 Subject Recruitment and Sample Collection

We collected urine samples from 64 patients with bladder cancer and 94 healthy volunteer controls from January 2018 to February 2020 at the Department of Urology, Peking University Shougang Hospital. The healthy volunteer controls were recruited through a hospital‐based volunteer program and were individuals without any history of bladder cancer or other urological conditions. Both bladder cancer patients and healthy volunteer controls fulfilled the following inclusion criteria: (1) no prior antibiotic use within the past month and (2) no history of sexually transmitted infections, obesity, diabetes, or recent symptoms of urinary tract infections. Exclusion criteria included individuals with conditions that could confound the microbiota composition, such as chronic inflammatory diseases or immunosuppressive disorders. The healthy volunteer controls were specifically selected to ensure that their health status was unrelated to the medical conditions being studied in the bladder cancer patients. The study was approved by the institutional review board of Peking University Shougang Hospital (IRBK‐2017‐053‐09). Written informed consent was obtained from all participants for inclusion in this study. Clean‐catch midstream urine (40 mL) was collected from all participants and centrifuged at 4°C, 2000 g, for 10 min to obtain a pellet, which was stored at −80°C until DNA extraction. Detailed clinical characteristics of the participants are provided in Table 1.

**Table 1:** Overview of the participants' characteristics.

	Healthy	Bladder cancer
Numbers of participants	94	64
Sex		
Men	41	58
Women	53	6
Median age (years)		
Men	53 (40–81)	67 (43–95)
Women	56 (43–80)	68.5 (60–78)
Disease status	N/A	
Primary tumor		45
Recurrent tumor		19
TNM Stage	N/A	
Ta		31
T1		16
T1 + Tis		1
T2		12
T3		4
Malignancy grade (H:L)	N/A	
High		43
Low		21
Risk (H:I:L)	N/A	
High		39
Intermediate		10
Low		15

*Note:* N/A, not applicable (e.g., disease‐related information for healthy individuals).

### 2.2 DNA Extraction and 16S rRNA Gene Sequencing

DNA was extracted from the urine pellet sediments using the Tianamp Micro DNA Kit (Tiangen) following the manufacturer's instructions. DNA concentrations were measured using a Qubit fluorometer (Invitrogen) to standardize input. The V4 region of the 16S rRNA gene was amplified using the 515F/806 R fusion primers (515 F: 5′‐GTGCCAGCMGCCGCGGTAA‐3′ and 806 R: 5′‐GGACTACHVGGGTWTCTAAT‐3′). PCR products were separated by electrophoresis in 2% agarose gels, purified using the QIAquick Gel Extraction Kit (Qiagen), and quantified using a Qubit fluorometer. Purified DNA (20 ng) was adjusted to a consistent concentration to ensure equal DNA input for sequencing. Sequencing was performed on the BGISEQ‐500 platform using the single‐end 400 bp (SE400) module. The sequencing data are available in the China National GeneBank Nucleotide Sequence Archive (https://db.cngb.org/cnsa; accession number CNP0004972) [28, 29].

### 2.3 Data Processing

Raw sequences were trimmed to remove adapters and primers using Cutadapt (version 1.18) [30]. BWA mem (version 0.7.15‐r1140) [31] was used to remove sequences blasted to the hg19 reference genome to obtain sequences belonging only to bacteria. The remaining sequences were quality‐filtered, dereplicated, and checked for chimeras using VSEARCH (version 2.14.1) [32]. Operational taxonomic units (OTUs) were clustered at 97% similarity using UPARSE [33], and representative OTU sequences were taxonomically assigned using the syntax command in VSEARCH against the Greengenes database [34] with a 97% confidence estimate—a prevalence filter removed spurious OTUs observed in less than 10% of the samples. Samples were randomly resampled with the minimum sequences using the rarefy_even_depth (rngseed = TRUE) function from the phyloseq package in R, and the resampled OTU table was then used for further analysis.

### 2.4 Bioinformatics and Statistical Analysis

Bioinformatics and statistical analyses were carried out using the package vegan (v.2.6‐4) when not specified. Alpha diversity was evaluated as Chao1, richness (S′), the Simpson index (D′), Shannon diversity (H′), and Pielou's evenness (J′) using alpha_div command of USEARCH (version 10.0.240) [35]. Among these factors, richness is the number of observed OTUs, and along with Chao1, an indicator of species richness. Both the Simpson index and the Shannon diversity index are indicators of species diversity. Pielou's evenness is an indicator of community evenness. Principal coordinate analysis (PCoA) was performed using the unweighted UniFrac distances to compare the microbial composition within subgroups.

Three complementary nonparametric multivariate analyses based on the unweighted UniFrac distance, the multiresponse permutation procedure (MRPP) [36], analysis of similarity (ANOSIM) [37], and nonparametric multivariate analysis of variance (Adonis) [38] were used to test the differences in urinary microbial communities between subgroups. Variance partition analyses [39] were used to assess and compare the impact of participant characteristic variables on urinary microbial communities in each group.

The molecular ecological networks of different groups were constructed on the basis of the methods of random matrix theory (RMT) and the steps of network construction have been described in a previous report [40]. In the present study, only the OTUs in at least half of the total samples were retained for network construction. All network analyses were performed in the Molecular Ecological Network Analysis Pipeline developed by the Institute of Environmental Genomics, University of Oklahoma (http://ieg4.rccc.ou.edu/MENA/). Network visualization was executed using Gephi (version 0.9.7) [41] to demonstrate associations between species in urinary microbiota, and modules in each network were randomly colored.

Phylogenetic Investigation of Communities by Reconstruction of Unobserved States (PICRUSt) [42] against KEGG categories was performed to predict the functional pathways of urinary microbiota. The functional profiles were input into linear discriminant analysis effect size (LEfSe) [43], and linear discriminant analysis (LDA) was used to identify significantly different functional pathways between groups. Only the pathways with a logarithmic LDA score greater than 2 at *p* < 0.05 were considered significantly different.

We used a Wilcoxon rank‐sum test to determine significant differences in sample age, high‐quality clean reads, diversity, and relative abundance between subgroups. In light of previous work showing that age is significantly associated with urine microbiome [18], the *p‐*values of diversity, relative abundance, and pathways were correlated with diagnosis using semipartial Spearman correlation tests (R package ppcor) adjusting for age. Moreover, all *P* values for relative abundance were adjusted for multiple comparisons using Storey's false discovery rate (FDR) method.

## 3 Results

### 3.1 Sample Characteristics and 16S rRNA Gene Sequencing Data Summary

**Figure 1:**
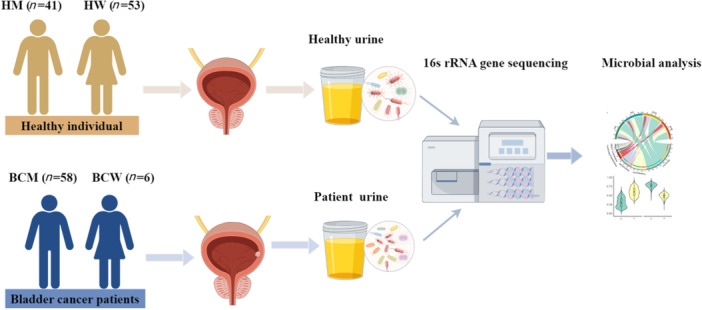
Flow diagram of the study. Urine samples were collected from healthy individuals and patients diagnosed with bladder cancer. These samples were subsequently subjected to 16S rRNA gene sequencing, and the resulting data were analyzed to characterize the urinary microbiome comprehensively.

We included 158 participants in this study, with 94 healthy controls (41 men and 53 women) and 64 bladder cancer patients (58 men and 6 women) (Figure 1). The bladder cancer group included 45 primary tumors (70.3%) and 19 recurrent tumors (29.7%) (Table 1). Furthermore, a single female primary tumor was classified as NMIBC. Among the primary tumors in men, 35 NMIBC cases and nine MIBC cases were identified. Recurrent tumors in women consisted of three NMIBC cases and two MIBC cases, whereas recurrent tumors in men included nine NMIBC cases and five MIBC cases. More detailed clinical information for each sample can be found in Supporting Information S1. The median ages of healthy men (HM), healthy women (HW), men with bladder cancer (BCM), and women with bladder cancer (BCW) were 53 (range, 40–81 years), 56 (range, 43–80 years), 67 (range, 43–95 years) and 68.5 (range, 60–78 years), respectively. Age was significantly greater in the bladder cancer group than in the healthy control group (women: *p* = 0.010, men: *p* = 1.4 × 10^−7^, Wilcoxon rank‐sum test; Supporting Information S1: Figure S1a).

A total of 405,492,490 raw reads were generated from 158 samples, resulting in 104,841,153 high‐quality reads (mean per sample = 663,552 ± standard deviation 302,674 reads) after adapter and primer trimming, host sequencing removal, quality filtering, and OTU prevalence (Supporting Information S1: Table S1). Significant differences in the number of clean reads were detected between the BCM and HM groups (*p* = 0.022, Wilcoxon rank‐sum test; Supporting Information S1: Figure S1b). Thus, each sample was randomly resampled with 37,930 sequences (the minimum sequences) to address disparities in sequence depths, and 14% of the sequences could be classified at the species level, 81% at the genus level, 89% at the family level, and 96% at the phylum level or higher taxa (Supporting Information S1: Table S2). Rarefaction analysis indicated sufficient sequencing depth for all samples (Supporting Information S1: Figure S2).

### 3.2 Urinary Microbiota Diversity and Composition in Bladder Cancer and Healthy Controls

**Figure 2:**
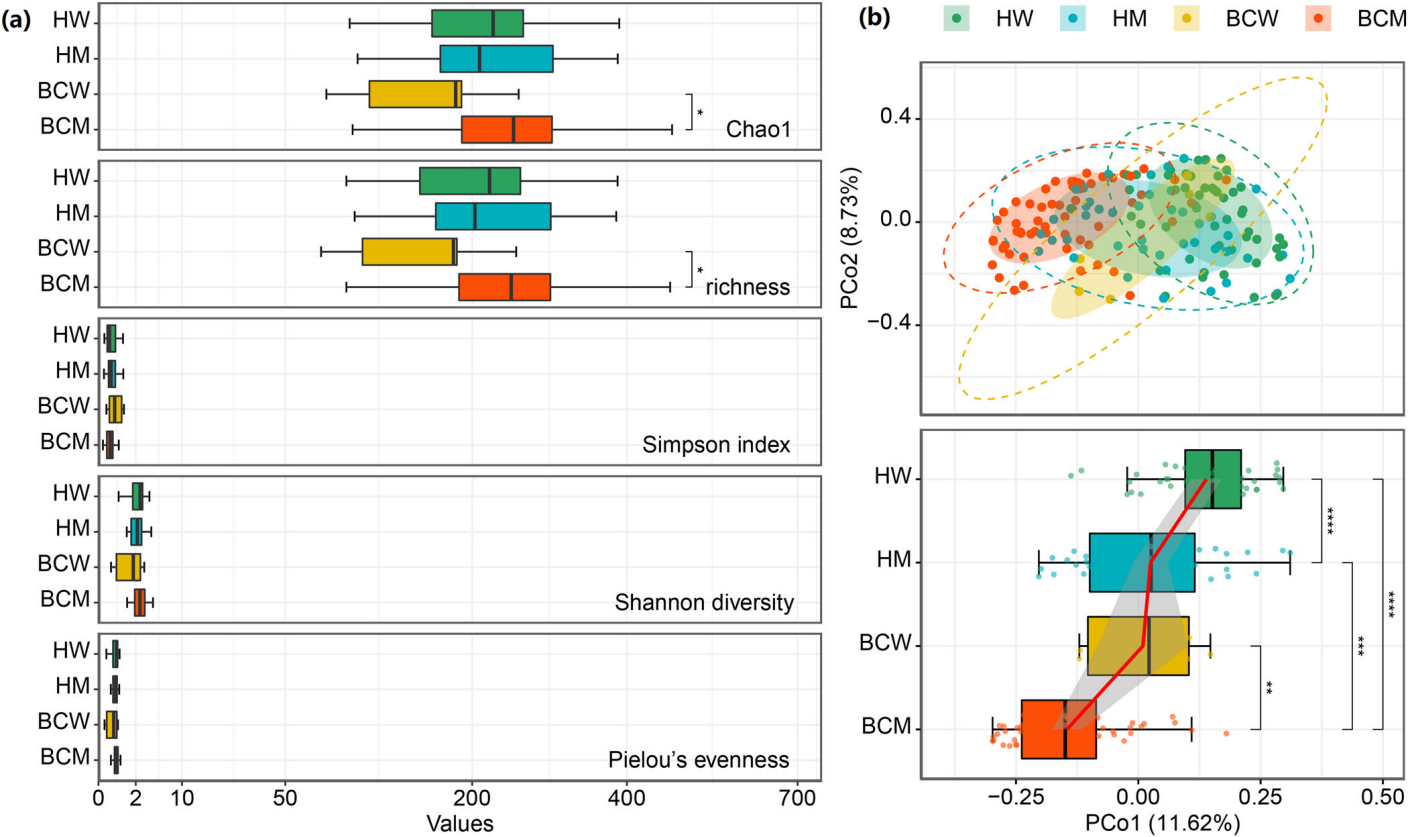
Alpha diversity and microbial community compositions in different subgroup samples. (a) Alpha diversity indices in four groups: healthy women (HW; *n* = 53, green), healthy men (HM; *n* = 41, blue), women with bladder cancer (*n* = 6, yellow), and men with bladder cancer (*n* = 58, orange), as shown in the boxplot (left panel). The indices include Chao1, richness, the Simpson index, the Shannon diversity index, and Pielou's evenness. (b) Individual urinary microbiota compositions in groups plotted on an unweighted UniFrac PCoA plot (upper right panel). The shaded ellipses represent the 68% confidence interval, and the dotted ellipse borders represent the 95% confidence interval. Urine samples collected from men with bladder cancer were distinguishable from healthy samples (HW: *p* = 1.00e^−11^, HM: *p* = 1.15e^−04^, Wilcoxon rank‐sum test and diagnosis using a semipartial Spearman correlation test for age), whereas samples from women were distinguishable from those from men (cancer: *p* = 6.42e^−03^, healthy: *p* = 4.31e^−05^, Wilcoxon rank‐sum test and diagnosis using a semipartial Spearman correlation test for age) (right‐down panel). *p < 0.05, **p < 0.01, ***p < 0.001, ****p < 0.0001.

The urinary microbiota richness differed across the four subgroups analyzed. Species richness was the highest in BCM (Chao1 = 251.04 ± 90.44, S′ = 247.48 ± 91.04), followed by HM (Chao1 = 241.94 ± 99.92, S′ = 238.20 ± 100.31), then by HW (Chao1 = 226.06 ± 92.61, S′ = 222.11 ± 93.16), and the lowest in BCW (Chao1 = 161.22 ± 68.58, S′ = 157.50 ± 69.26; Figure 2a). Species richness was significantly lower in BCW than in BCM (Chao1: *p* = 0.021, richness: *p* = 0.020, Wilcoxon rank‐sum test and diagnosis using a semipartial Spearman correlation test for age), which might be due to the small sample size in BCW. Both Shannon diversity and Pielou's evenness were greatest in BCM (H′ = 2.47 ± 0.79, J′ = 0.45 ± 0.12), followed by HW (H′ = 2.30 ± 0.83, J′ = 0.43 ± 0.14), HM (H′ = 2.16 ± 0.79, J′ = 0.40 ± 0.13), and BCW (H′ = 1.62 ± 1.20, J′ = 0.31 ± 0.22). Compared with that of BCM, species diversity in the urinary microbiota of HM decreased (*p* = 0.044, Wilcoxon rank‐sum test), as did Pielou's evenness (*p* = 0.045, Wilcoxon rank‐sum test). After adjusting for age, these differences between HM and BCM groups were no longer significant.

**Figure 3:**
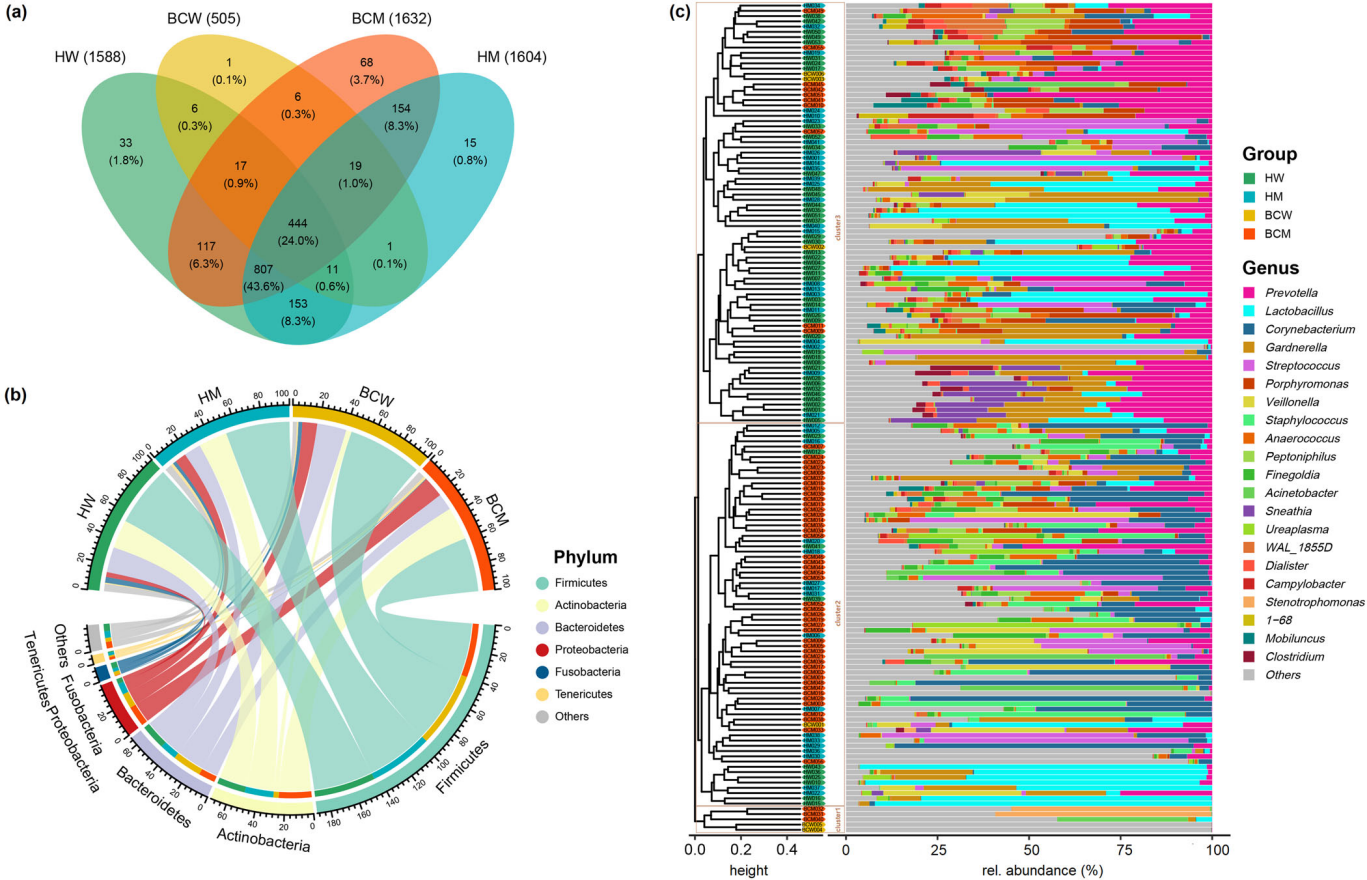
Operational Taxonomic Unit (OTU) Venn diagram and relative abundance. (a) Venn diagram showing the number of OTUs shared or not shared by subgroups. (b) Chord diagram displaying the average relative abundance (%) of the urinary microbiota at the phylum level. The scale indicates total abundance and the width of each chord represents the abundance of each phylum in different subgroup samples. (c) Hierarchical clustering of individual urinary microbiota at the genus level. The clustering of samples was performed on the basis of the unweighted UniFrac distance using the complete clustering method. Unclassified taxa and taxa with a mean relative abundance < 0.5% are grouped as “Others.”

Differences in microbial composition among the four subgroups were observed in the unweighted UniFrac PCoA plots (Figure 2b, top panel). The microbiota of individuals, when plotted by subgroup, revealed prominent features: Microbiota from the men with bladder cancer were distinguishable from those from HM (HW: *p* = 1.00e^−11^, men: *p* = 1.15e^−04^, Wilcoxon rank‐sum test and diagnosis using a semipartial Spearman correlation test for age; Figure 2b, bottom panel), and women were distinguishable from men both in the cancer (*p* = 6.42e^−03^, Wilcoxon rank‐sum test) and healthy groups (*p* = 4.31e^−05^, Wilcoxon rank‐sum test and diagnosis using a semipartial Spearman correlation test for age). For samples from patients of the same sex, no significant differences in diversity were found between primary and recurrent tumors (Supporting Information S1: Figure 3).

At the 97% similarity level, 1852 OTUs were obtained, and the majority of OTUs were found in BCM (1632 OTUs), followed by HM (1604 OTUs), HW (1588 OTUs), and BCW (505 OTUs) (Figure 3a). Only 24.0% of OTUs were shared across all four subgroups, and the percentage of OTUs shared by the other subgroups reached 67.6%, except for BCW. There were 33 identifiable OTUs in the HW group, 15 in the HM group, one in the BCW group, and 68 in the BCM group. The urinary microbiota contained 13 phyla and 158 genera in total, and the most abundant phyla were Firmicutes, Actinobacteria, Bacteroidetes, Proteobacteria, Fusobacteria, and Tenericutes (Figure 3b).

The most common genera of all the samples were *Prevotella, Lactobacillus*, *Corynebacterium*, and others, as shown in Figure 3c. Notably, the genera *WAL_1855D* and *1‐68*, belonging to the Tissierellaceae family, were present. Clustering analysis of the urinary microbiota revealed three distinct clusters based on the unweighted UniFrac distance. Cluster 2 was enriched in the bladder cancer samples (46 bladder cancer patient samples vs. 27 healthy controls), with *Corynebacterium* being dominant (average relative abundance: 17.3%). Cluster 3 was enriched in healthy controls (13 bladder cancer patient samples vs. 67 healthy controls; *p* = 2.72e^−9^, Fisher's exact test with 95% CI), with the genus *Prevotella* dominating (average relative abundance: 16.3%). Cluster 1 showed some inconsistencies, containing two BCW samples dominated by OTU1 from the Lactobacillales order (relative abundance > 96%) and three BCM samples, two of which were dominated by the genus *Stenotrophomonas* (relative abundance > 50%) and one by the family Enterobacteriaceae (relative abundance: 48.3%). Because of the significantly lower number of OTUs and the scattered clustering, the urinary microbiota from the six BCW samples were deemed unrepresentative and excluded from further community structure analysis.

We performed three nonparametric multivariate statistical tests (MRPP, ANOSIM, and Adonis) to explore the relationships between microbiome composition and other variables. These tests revealed that urinary microbiome composition was significantly associated with age, sex, and disease status across all the samples (Table 2). Additionally, a slight difference was detected in the MRPP in the urine microbiome between primary tumors and recurrent tumors. Statistical tests were also conducted on primary and recurrent tumor samples to explore associations between microbiome composition and type, malignancy grade, or risk. MRPP and Adonis detected a significant relationship between the urine microbiome of primary tumors and type.

**Table 2:** Significance tests on the effects of the participants' characteristics on the urinary microbial community structure.

Data sets	*n*	MRPP		ANOSIM		Adonis	
		*δ*	*p*	*R*	*p*	*F*	*p*
Age	129	0.670	0.004	0.063	0.078	4.379	0.001
Sex	152	0.665	0.001	0.203	0.001	7.980	0.001
Disease status	152	0.660	0.001	0.238	0.001	10.300	0.001
Primary vs. recurrence	58	0.638	0.040	0.053	0.261	1.369	0.068
Type (MIBC vs. NMIBC)	58	0.640	0.138	0.083	0.165	1.271	0.094
Primary men	44	0.631	0.004	0.060	0.309	1.872	0.004
Recurrence men	14	0.632	0.160	0.252	0.063	1.504	0.075
Malignancy grade (H:L)	58	0.640	0.158	−0.008	0.514	1.199	0.161
Primary men	44	0.637	0.441	−0.012	0.553	1.011	0.417
Recurrence men	14	0.646	0.643	−0.158	0.571	0.854	0.628
Risk (H:I:L)	58	0.640	0.255	0.003	0.432	1.076	0.279
Primary men	44	0.637	0.359	0.001	0.490	1.039	0.351
Recurrence men	14	0.649	0.700	−0.127	0.608	0.802	0.796

*Note:* Three different permutation tests were performed using the unweighted UniFrac distance, including the multi‐response permutation procedure (MRPP), analysis of similarities (ANOSIM), and permutational multivariate analysis of variance (Adonis).

### 3.3 Specific Differences and Functional Pathways in Healthy Urinary Microbiota Between Men and Women

The phylum Synergistetes was more abundant in women (HW vs. HM: 39.6% (21/53) vs. 14.6% (6/41), *p* = 0.0060), whereas Thermi were more abundant in men (HW vs. HM: 2 of 53 vs. 9 of 41, *p* = 0.0066), based on a Wilcoxon rank‐sum test, followed by Storey's FDR method (*p* < 0.05 and FDR < 0.05, Supporting Information S1: Table S3). After adjusting for age, these differences remained significant. At the genus level, *Acinetobacter*, *Chryseobacterium*, and *Lactobacillus* were significantly more abundant in women, whereas *Corynebacterium*, *Staphylococcus*, and *Veillonella* were more abundant in men.

A total of 176 pathways were predicted from all samples, with notable pathways including amino acid metabolism, carbohydrate metabolism, lipid metabolism, metabolism of cofactors and vitamins, metabolism of terpenoids and polyketides, and xenobiotic biodegradation and metabolism (Supporting Information S1: Table S7). In HW, 23 pathways were significantly more abundant, whereas 24 pathways were more abundant in HM (LDA > 2, *p* < 0.05, and *p* (spcor, AGE) < 0.05; Supporting Information S1: Figure S4).

### 3.4 Differential Abundance and Functional Pathways of Urinary Microbiota Between Cancer Patients and Healthy Individuals

**Figure 4:**
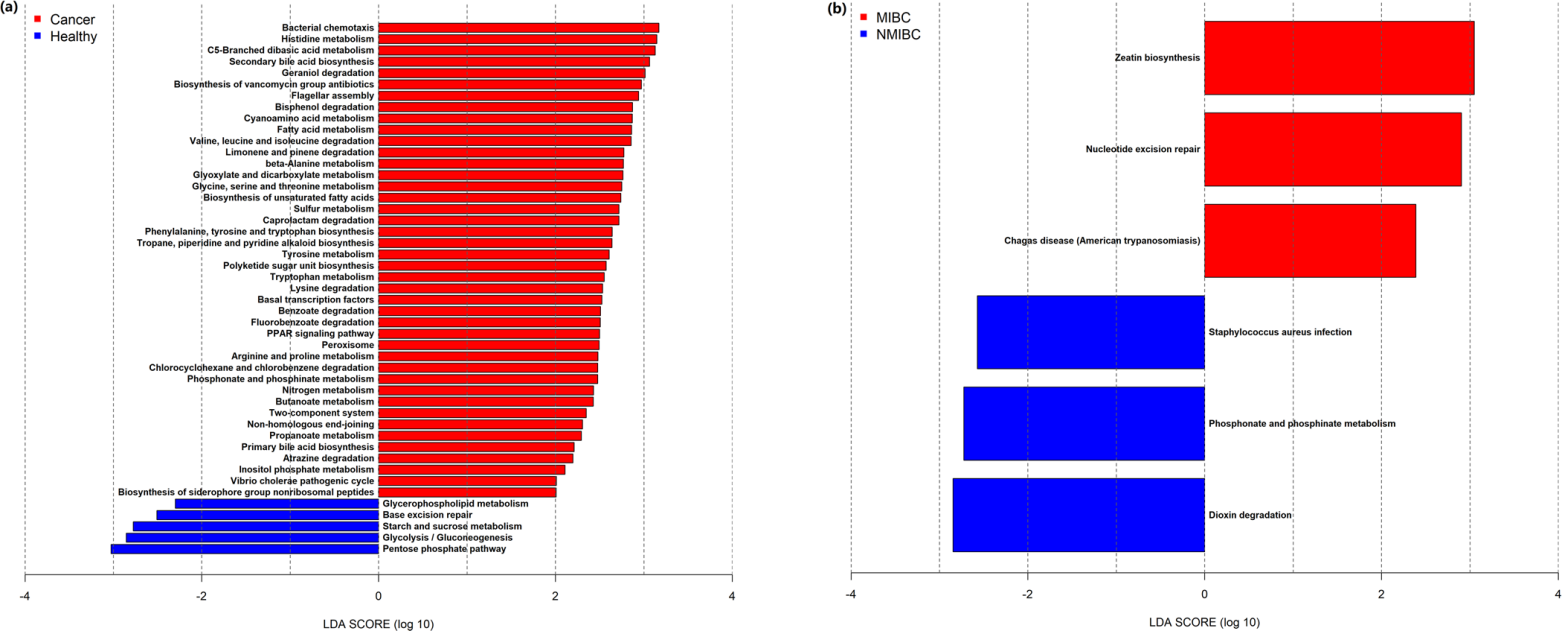
Predicted functional analysis. (a, b) KEGG pathways were significantly differentially enriched between healthy men (HM; *n* = 41, blue) and men with bladder cancer (*n* = 58, red), as well as NMIBC (*n* = 35, blue) and MIBC (*n* = 9, red) samples.

At the phylum level, Cyanobacteria (BCM vs. HM: 43 of 58 vs. 21 of 41) and Synergistetes (BCM vs. HM: 26 of 58 vs. 6 of 41) were differentially more abundant in men with bladder cancer, whereas Fusobacteria (BCM vs. HM: 44 of 58 vs. 38 of 41) were more abundant in HM (*p* < 0.05, FDR < 0.05 and *p* (spcor, AGE) < 0.05, Supporting Information S1: Table S4). At the genus level, the abundances of *Acinetobacter*, *Propionibacterium*, *Staphylococcus*, and *Stenotrophomonas* were significantly greater in men with bladder cancer, whereas *Gardnerella*, *Gemella*, *Helcococcus*, *Lactobacillus*, and *Sneathia* were more abundant in HM. Forty‐two pathways were significantly enriched in men with bladder cancer, with amino acid metabolism being the most notable (LDA > 2, *p* < 0.05, and *p* (spcor, AGE) < 0.05, Figure 4a). In HM, five other pathways were significantly enriched, and the most notable pathway was carbohydrate metabolism, which included glycolysis/gluconeogenesis, starch and sucrose metabolism, and the pentose phosphate pathway.

### 3.5 Differential Abundance and Functional Pathways of Urinary Microbiota Between Primary NMIBC and MIBC

**Figure 5:**
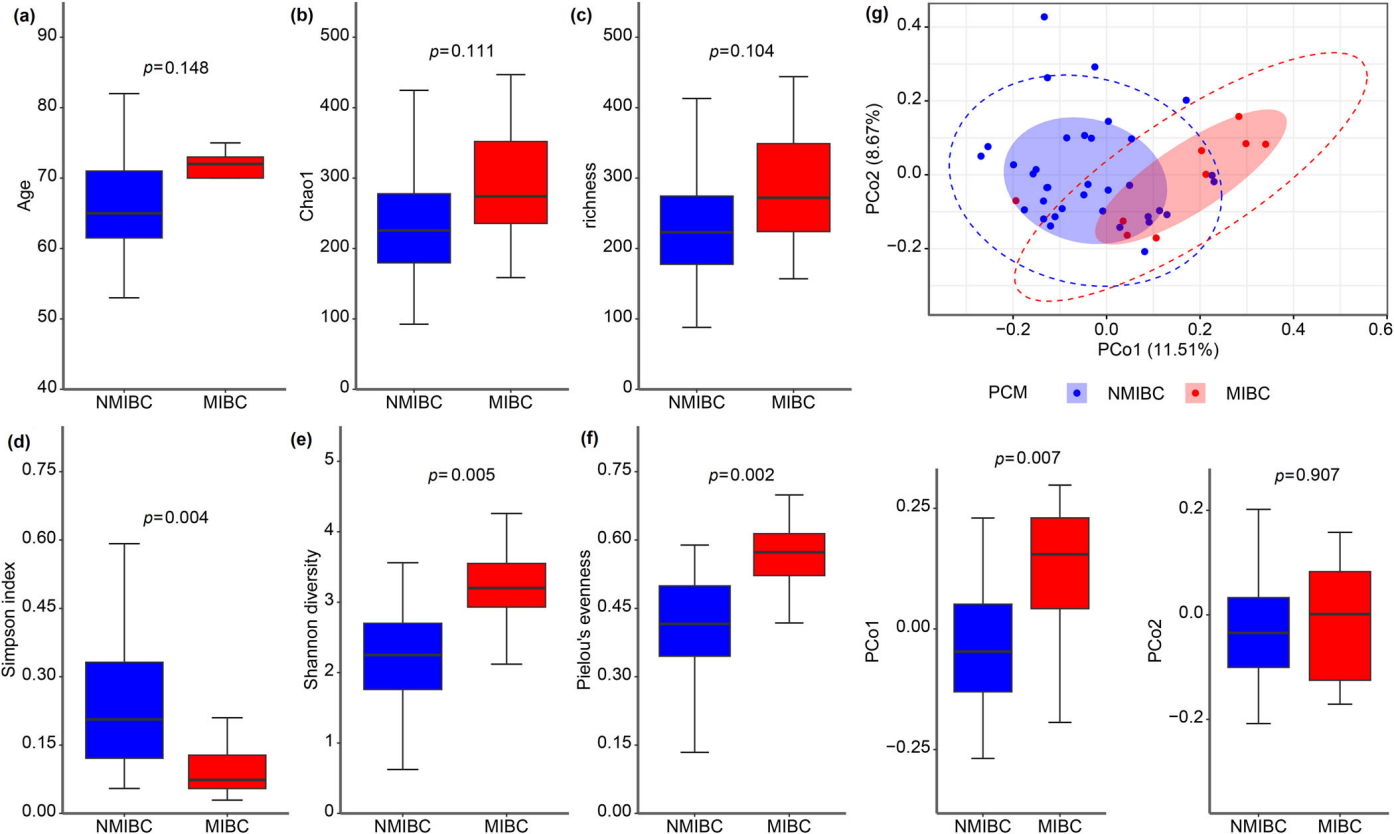
Alpha diversity and microbial community compositions for non‐muscle‐invasive bladder cancer (NMIBC) and muscle‐invasive bladder cancer (MIBC) of primary bladder cancer in men (PCM). (a–f) Boxplot showing age and alpha diversity in the NMIBC (*n* = 35, blue) and MIBC (*n* = 9, red) groups (a, age; b, Chao1 index; c, richness index; d, Simpson index; e, Shannon diversity index; f, Pielou's evenness index). (g) Individual urinary microbiota compositions in groups plotted on an unweighted UniFrac PCoA plot (upper right panel). Urine samples collected from the patients with NMIBC differed significantly from those from patients with MIBC in PCo1 (*p* = 0.007, Wilcoxon rank‐sum test and diagnosis using a semipartial Spearman correlation test for age, right‐down panel).

Although age and urinary microbiota species richness metrics (Chao1 and richness) did not differ between primary NMIBC and MIBC, a significantly lower Simpson index, higher Shannon diversity, and Pielou's evenness were presented in MIBC than in NMIBC, indicating a greater species diversity in the urinary microbiota (D′: *p* = 0.004, H′: *p* = 0.005, J′: *p* = 0.002, Wilcoxon rank‐sum test and diagnosis using a semipartial Spearman correlation test for age; Figure 5a–f). Differences in microbial composition between primary NMIBC and MIBC were observed in the unweighted UniFrac PCoA plots (Figure 5g, top panel). The microbiota from patients with primary MIBC were significantly greater than those from patients with NMIBC (*p* = 0.007, Wilcoxon rank‐sum test and diagnosis using a semipartial Spearman correlation test for age; Figure 5b, bottom panel). Fusobacteria (NMIBC vs. MIBC; 27 of 35 vs. 9 of 9, *p* = 0.0079) were differentially enriched in primary MIBC (*p* < 0.05, FDR < 0.05, and *p* (spcor, AGE) < 0.05, Supporting Information S1: Table S6). At the genus level, *1‐68* and *Peptoniphilus* were enriched in primary MIBC. Phosphonate and phosphinate metabolism, dioxin degradation, and *Staphylococcus aureus* infection significantly enriched pathways in patients with primary NMIBC, belonging to the metabolism of other amino acids, xenobiotic biodegradation and metabolism, and bacterial infectious disease, respectively. (LDA > 2, *p* < 0.05, and *p‐* (spcor, AGE) < 0.05, Figure 4b). Zeatin biosynthesis and nucleotide excision repair, which are parasitic infectious diseases, terpenoid and polyketide metabolism, replication, and repair were significantly enriched in primary MIBC.

### 3.6 RMT‐Based Molecular Ecological Networks

**Figure 6:**
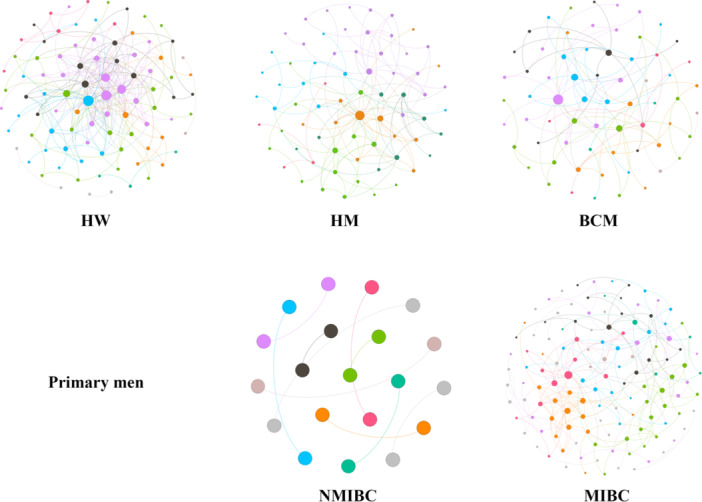
Molecular ecology networks for urinary bacterial communities. Each node represents an OTU. The edge indicates a strong and significant (*p* < 0.01) correlation between two nodes. The colors of the nodes indicate the different modules, and the size of each node is proportional to the number of connections.

On the basis of the 16S rRNA gene sequencing data, RMT‐based molecular ecological networks were used to analyze potential microbe–microbe interactions in urinary microbiota communities. The network of urinary microbiota communities differed between the sexes (HW vs. HM), disease statuses (HM vs. BCM), and disease types (NMIBC vs. MIBC in Figure 6). Networks were simpler in HM than in HW but more connected and complex in contrast with bladder cancer men. Although there were only a small number of samples, the networks in primary MIBC samples were more complicated than those in primary NMIBC. The similar variation of networks was also indicated in the topological properties (Table 3). Compared with those in HW, the number of total nodes, total links, and average degree decreased in HM, whereas they increased compared with those in men with bladder cancer, indicating the increased network complexity. For primary MIBC, the urinary microbiota network contained 200 links among 130 nodes, which was much greater than the number of networks of primary NMIBC (10 links among 20 nodes), reflecting a greater number of microbial co‐occurrences in the urinary microbiota community.

**Table 3:** Topological properties of the molecular ecological networks of urinary microbial communities in different groups.

Network indexes	HW53(0.580)	HM41(0.580)	BCM_58(0.580)	NMIBC35(0.830)	MIBC9(0.840)
Total nodes	92	71	66	20	130
Total links	233	129	90	10	200
R square of power‐law	0.921	0.839	0.772	0	0.785
Average degree (avgK)	5.065	3.634	2.727	1	3.077
Average clustering coefficient (avgCC)	0.06	0.066	0.092	0	0.157
Harmonic geodesic distance (HD)	2.647	2.747	3.226	1	3.936
Efficiency	0.944	0.957	0.966	0	0.977

## 4 Discussion

In the present study, we analyzed the urinary microbiota of 64 patients with bladder cancer and 94 disease‐free controls using 16S rRNA gene sequencing. Our findings indicate that although the overall microbial diversity in the bladder remains relatively consistent, changes in microbial composition can be influenced by factors such as sex, disease status, and disease progression. Notably, significant differences in both alpha and beta diversity were observed between the NMIBC and MIBC groups. The identification of *Peptoniphilus* spp. as a bacterium that differed significantly between the two groups highlights its potential role in the progression of bladder cancer.

Our present research encompassed a broader range of variables, including sex, age, and disease status, which increased the complexity of analyzing the urine microbiome in patients with bladder cancer. Previous studies have typically focused on more homogeneous cohorts, often consisting of male participants of similar age. Although such homogeneous samples help control for potential confounding variables, they also limit the universality of the findings to a broader patient population. Our nonparametric multivariate statistical analysis confirmed significant relationships between the urine microbial community and factors such as age, sex, and disease status (Table 2). These results emphasize the importance of considering these variables in future studies to understand better the microbial differences associated with bladder cancer. We recommend that multivariate analysis be performed before microbial difference analysis to account for the interactions between multiple variables, providing a more reliable assessment of the relationship between the urinary flora and bladder cancer.

In the present study, we observed differences in alpha and beta diversity between the NMIBC and MIBC groups and identified an enrichment of *Peptoniphilus* spp. in the MIBC group compared with the NMIBC group. The genus *Peptoniphilus* is a gram‐positive anaerobic coccus associated with opportunistic infections, particularly urinary tract infections and chronic wounds. The genus has been isolated from various clinical samples, including blood, urine, and amniotic fluid [44, 45]. We hypothesize that *Peptoniphilus* spp. may play a role in bladder cancer progression by inducing persistent inflammation, which is a known driver of tumorigenesis. Chronic inflammation is a critical factor in the development and progression of cancer, as it increases the risk of harmful mutant cells accumulating in the host and can lead to tissue damage through the excessive release of cytotoxic enzymes, reactive oxygen species, and matrix metalloproteinases (MMPs) [46, 47, 48]. Increased MMP expression is associated with invasive bladder cancer phenotypes and plays a key role in tumor progression [49]. Thus, *Peptoniphilus* spp. may indirectly promote cancer progression by exacerbating inflammation and facilitating extracellular matrix degradation, which contributes to tumor invasion and metastasis. On the basis of these mechanisms, *Peptoniphilus* spp. may serve as a potential biomarker for risk stratification in bladder cancer, particularly for distinguishing between NMIBC and MIBC.

Our present study also contributes to the growing body of research on the potential therapeutic implications of the urinary microbiota in bladder cancer. For example, bacillus Calmette‐Guerin (BCG) therapy, which is commonly used to treat intermediate‐ and high‐risk NMIBC, has shown efficacy in reducing tumor recurrence and progression [50]. Although the role of the urinary microbiota in bladder cancer treatment remains underexplored, studies in other cancers, such as melanoma, have demonstrated that the tumor‐associated microbiome can influence the effectiveness of immunotherapy [51]. Thus, our findings on the urinary microbiota in NMIBC and MIBC patients may offer novel insights for developing microbiome‐based interventions for bladder cancer treatment.

A reduction in microbial diversity is a well‐documented feature of diseases such as Crohn's disease, ulcerative colitis, and colorectal cancer [52]. After adjusting for age, we did not observe a significant reduction in urinary microbial alpha diversity between bladder cancer patients and healthy controls (Figure 2a). However, our beta‐diversity analysis revealed differences in microbial composition among the four subgroups (Figure 2b). Clustering analysis of the urine microbiota at the genus level revealed that cancer and control groups formed separate clusters, with the cancer group showing reduced proportions of beneficial genera such as *Lactobacillus* and *Prevotella* and an increased abundance of urinary tract pathogens such as *Corynebacterium* and *Acinetobacter*.

Further investigation of specific bacterial genera revealed that *Stenotrophomonas*, *Propionibacterium*, *Acinetobacter*, and *Staphylococcus* were more abundant in patients with bladder cancer than in healthy controls (Supporting Information S1: Table S4). *Stenotrophomonas* spp., a gram‐negative, nonfermenting, and motile bacillus, was shown in a study by Ammar et al. to exert significant cytotoxic effects in bladder cancer patients, leading to the death of epithelial cells. This type of cell death can accelerate cell regeneration, increasing the risk of DNA replication errors and potentially contributing to the onset and progression of cancers, such as bladder cancer [53]. *Propionibacterium* spp., which are commonly found in the skin flora and mucosal surface of the urethra, can resist phagocytosis by macrophages through their complex cell walls. It also secretes extracellular enzymes that damage host tissues and other enzymes that may be involved in inflammatory infiltration and epithelial permeability [54, 55]. Several studies have linked *Propionibacterium* spp. to chronic inflammation and cancer pathogenesis, with their high prevalence in prostate cancer tissue samples being well documented [56, 57]. These findings suggest that *Stenotrophomonas* spp. and *Propionibacterium* spp. may initiate a pathogenic cascade that triggers bladder inflammation or invasion of epithelial cells, potentially contributing to cancer progression.

In addition to these genera, we observed increased abundance of *Acinetobacter* spp., *Staphylococcus* spp., and *Sphingomonas* spp. in patients with bladder cancer. These findings are consistent with previous studies showing a greater abundance of *Acinetobacter* spp. in bladder cancer tissues and *Staphylococcus* spp. in the urine of bladder cancer patients [19, 21]. *Sphingomonas* spp., which are often associated with nosocomial infections and are typically found in individuals with low immune function [58], were also more prevalent in our cancer group. Although the precise mechanisms by which these bacteria may influence bladder cancer remain unclear, these genera could play a role in the development and progression of the disease.

Our present analysis also revealed sex‐specific differences in the urinary microbiota. Consistent with previous literature reports, the genus *Lactobacillus* was abundant in women, whereas *Corynebacterium* spp., *Staphylococcus* spp., and *Veillonella* spp. were abundant in men [11, 59]. Interestingly, metabolic pathway enrichment analyses showed that the microbiota of women was enriched in pathways related to DNA replication and repair. By contrast, the microbiota of men was enriched in pathways associated with biodegradation and metabolism. These findings suggest that sex differences in urinary microbiota could contribute to the disparity in bladder cancer incidence between men and women.

We conducted a molecular ecological network analysis of the urinary microbiota, observing differences in network structure based on sex (HW vs. HM), disease status (HM vs. men with bladder cancer), and disease type (NMIBC vs. MIBC). We found that patients with high‐risk MIBC exhibited greater network complexity compared with NMIBC (Figure 6). This complexity suggests that cancer progression may alter the abundance, structure, and interactions within the urinary microbiota. However, how these changes affect microbial network complexity remains unclear. Further research is needed to explore the impact of these microbial interactions on bladder cancer progression.

Despite these insights, our study has several limitations. Although the clean‐catch midstream urine collection method avoids the discomfort associated with urethral catheterization, it may introduce contamination from the microbiota around the urethral orifice. Additionally, we lacked data on the urinary microbiota in patients with inflammation, which could confound cancer‐related microbiome changes. The small number of female patients with bladder cancer also limits our ability to determine sex‐specific microbial differences in bladder cancer. Further studies with larger, more balanced samples are needed to validate our findings and explore the potential mechanisms driving urinary microbiota alterations in patients with bladder cancer.

Despite its limitations, our study offers several strengths, including the use of more variables than previous studies and a more comprehensive analysis of urinary microbiota diversity and composition across different disease types. Our use of multivariate analysis provides valuable insights into how factors beyond the presence of tumors shape the urinary microbiota, and these findings may pave the way for future research on the role of the urinary microbiome in bladder cancer prevention and diagnosis.

## 5 Conclusions

In conclusion, our study revealed a potential relationship between urinary microbial disturbance and the development and progression of bladder cancer, and specific microorganisms may be related to disease status and risk stratification. Future research is needed to elucidate the mechanisms behind these associations and assess their clinical significance, offering new avenues for bladder cancer prevention and treatment.

## Author Contributions

**Nannan Li:** investigation (equal), methodology (equal), writing – original draft (lead). **Lei Wang:** investigation (equal), resources (equal), supervision (equal). **Qin Yang:** formal analysis (lead). **Fuqiang Li:** data curation (equal), supervision (equal). **Zhun Shi:** data curation (equal). **Xiujie Feng:** validation (equal). **Liwei Zhang:** investigation (equal). **Xiaojian Li:** investigation (equal). **Xin Jin:** writing – review and editing (equal). **Shida Zhu:** supervision (equal). **Kui Wu:** project administration (equal), supervision (equal), visualization (equal). **Ningchen Li:** project administration (equal), supervision (equal).

## Ethics Statement

The study was approved by the institutional review board of Peking University Shougang Hospital (IRBK‐2017‐053‐09).

## Consent

All samples were collected with written informed consent from adult participants.

## Conflicts of Interest

Qin Yang, Fuqiang Li, and Zhun Shi are the Project Scientists in BGI, Xin Jin is the Chief Scientist of BGI, Shida Zhu is the Deputy General Manager of BGI, and Kui Wu is Director Scientist of BGI. The authors declare that the research was conducted in the absence of any commercial or financial relationships that could be construed as potential conflicts of interest.

## Declaration of Generative AI and AI‐Assisted Technologies in the Writing Process

During the revision stage, AI tools were utilized to optimize 10% of the content expression, primarily focusing on paragraph structure and wording adjustments, while the core ideas and original content were independently developed by the author.

## Supporting information


**Supporting Figure 1.** Age of sample providers and high‐quality clean reads of the samples. (a) Provider age (HW vs. HM, *p* = 0.354; HW vs. BCW, *p* = 0.010; HW vs. BCM, *p* = 3.0e^−06^; HM vs. BCW, *p* = 0.002; HM vs. BCM, *p* = 1.4e^−07^; BCW vs. BCM, *p* = 0.881, Wilcoxon rank‐sum test) among subgroups. (b) Counts (HW vs. HM, *p* = 0.620; HW vs. BCW, *p* = 0.254; HW vs. BCM, *p* = 0.101; HM vs. BCW, *p* = 0.141; HM vs. BCM, *p* = 0.022; BCW vs. BCM, *p* = 0.845, Wilcoxon rank‐sum test) of high‐quality clean reads among subgroups. *p < 0.05, **p < 0.01, ***p < 0.001, ****: *p* < 0.0001.

**Supporting Figure 2.** Rarefaction curves. The x‐axis represents the number of randomly extracted sequences per sample and the y‐axis represents the number of observed operational taxonomic units (OTUs) based on the number of sequences. Each curve in the graph represents a different sample, and samples from different subgroups are shown in a different color. As the number of sequences increased, the number of observed OTUs also increased. Eventually, the curves began to plateau, indicating sufficient sequencing depth.

**Supporting Figure 3.** Age, alpha diversity, and microbial community compositions for primary and recurrent bladder cancer. (a–f) Boxplot showing age and alpha diversity in women with primary bladder cancer (*n*=1, blue), women with recurrent bladder cancer (*n* = 5, red), men with primary bladder cancer (*n* = 44, blue), and men with recurrent bladder cancer (*n* = 14, red) groups (a, age; b, Chao1 index; c, richness index; d, Simpson index; e, Shannon diversity index; f, Pielou's evenness index). (g) Individual urinary microbiota compositions in subgroups were plotted on an unweighted UniFrac PCoA plot (upper right panel). Urine samples collected from the primary bladder cancer patients were not significantly different from those collected from patients with recurrence (Wilcoxon rank‐sum test and diagnosis using a semipartial Spearman correlation test for age, right‐down panel).

**Supporting Figure 4.** Prediction functional analysis. KEGG pathways were significantly differentially enriched between healthy women (HW; *n* = 53, blue) and healthy men (HM; *n* = 41, red).

Supplementary Table‐final version.

## Data Availability

All data associated with this study are present in the paper or the Supporting Information.
