# Supplementary figures and images for: Identification and Evaluation of the Urinary Microbiota Associated With Bladder Cancer
Source: Cancer Innov. 2025 May 25;4(4):e70012. doi: 10.1002/cai2.70012 (PMC12103652; doi:10.1002/cai2.70012)

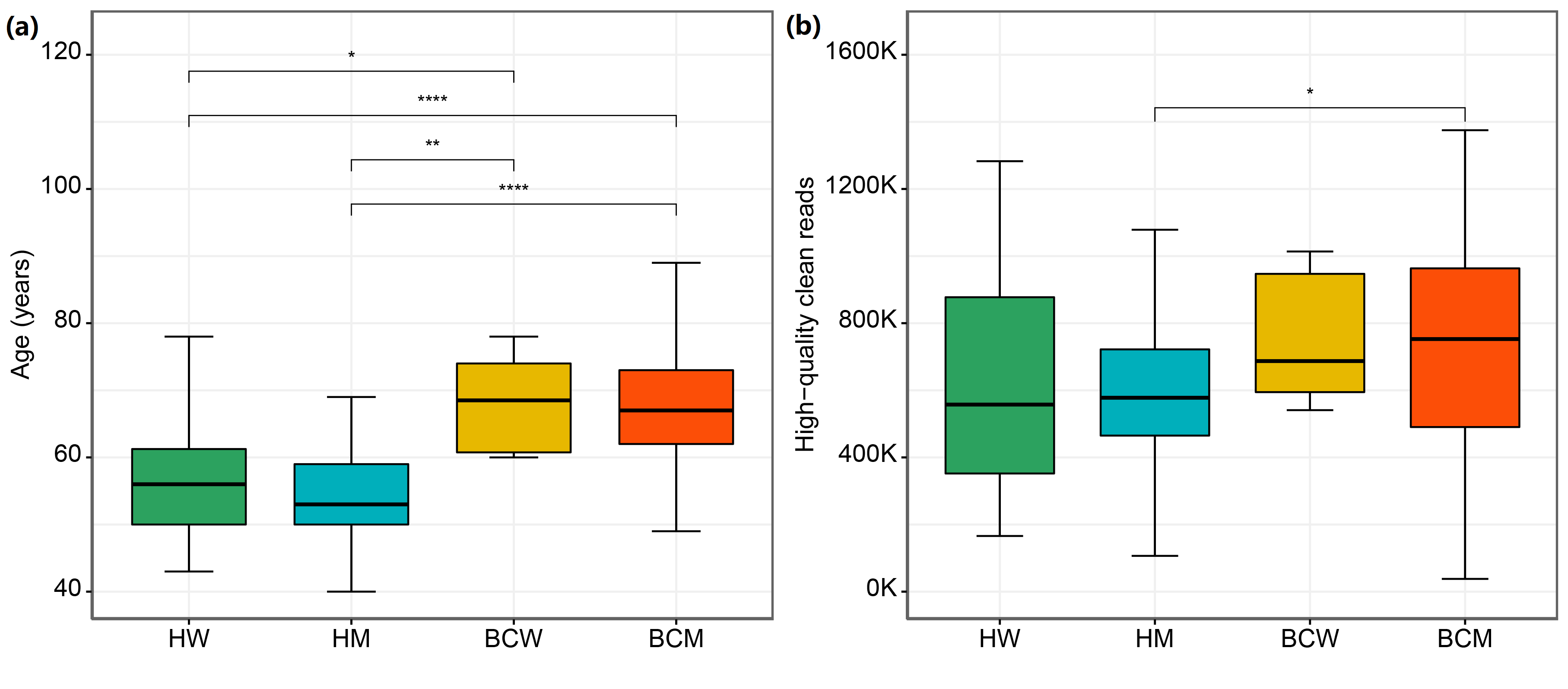

Supplement: Supplementary file 1 — Supporting Figure 1. Age of sample providers and high‐quality clean reads of the samples. (a) Provider age (HW vs. HM, p = 0.354; HW vs. BCW, p = 0.010; HW vs. BCM, p = 3.0e−06; HM vs. BCW, p = 0.002; HM vs. BCM, p = 1.4e−07; BCW vs. BCM, p = 0.881, Wilcoxon rank‐sum test) among subgroups. (b) Counts (HW vs. HM, p = 0.620; HW vs. BCW, p = 0.254; HW vs. BCM, p = 0.101; HM vs. BCW, p = 0.141; HM vs. BCM, p = 0.022; BCW vs. BCM, p = 0.845, Wilcoxon rank‐sum test) of high‐quality clean reads among subgroups. *p < 0.05, **p < 0.01, ***p < 0.001, ****: p < 0.0001. [file CAI2-4-e70012-s001.tif]

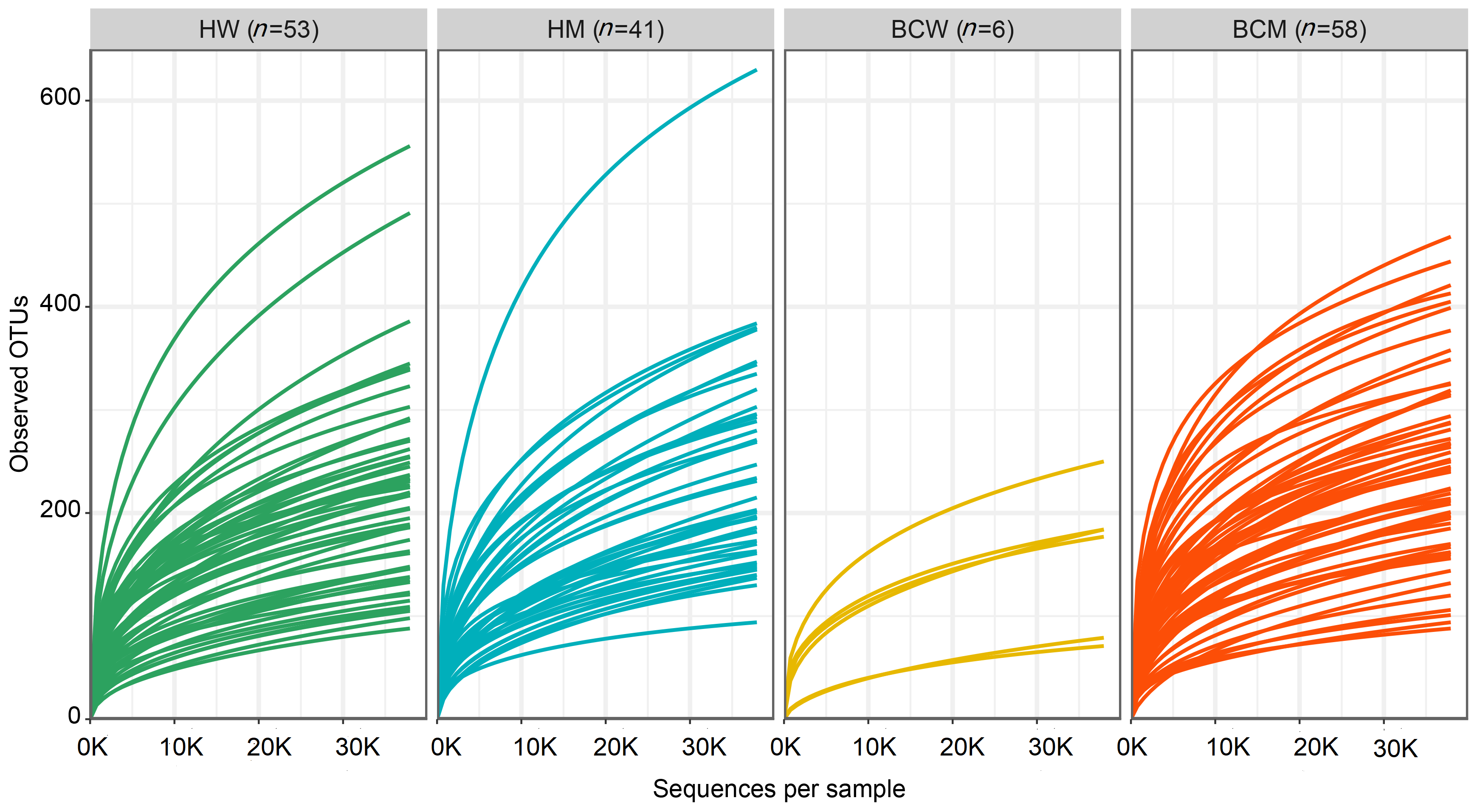

Supplement: Supplementary file 2 — Supporting Figure 2. Rarefaction curves. The x‐axis represents the number of randomly extracted sequences per sample and the y‐axis represents the number of observed operational taxonomic units (OTUs) based on the number of sequences. Each curve in the graph represents a different sample, and samples from different subgroups are shown in a different color. As the number of sequences increased, the number of observed OTUs also increased. Eventually, the curves began to plateau, indicating sufficient sequencing depth. [file CAI2-4-e70012-s005.tif]

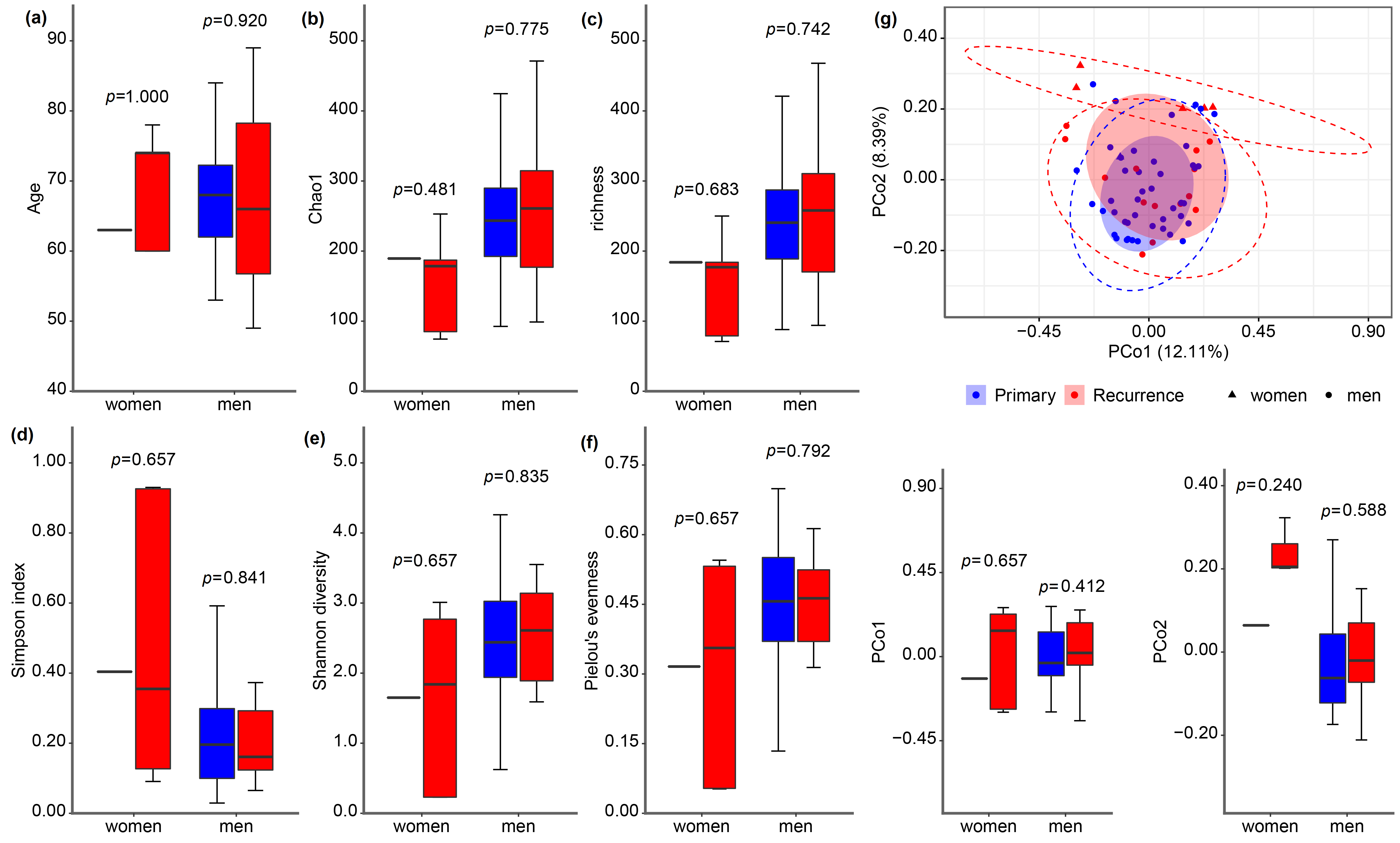

Supplement: Supplementary file 3 — Supporting Figure 3. Age, alpha diversity, and microbial community compositions for primary and recurrent bladder cancer. (a–f) Boxplot showing age and alpha diversity in women with primary bladder cancer (n=1, blue), women with recurrent bladder cancer (n = 5, red), men with primary bladder cancer (n = 44, blue), and men with recurrent bladder cancer (n = 14, red) groups (a, age; b, Chao1 index; c, richness index; d, Simpson index; e, Shannon diversity index; f, Pielou's evenness index). (g) Individual urinary microbiota compositions in subgroups were plotted on an unweighted UniFrac PCoA plot (upper right panel). Urine samples collected from the primary bladder cancer patients were not significantly different from those collected from patients with recurrence (Wilcoxon rank‐sum test and diagnosis using a semipartial Spearman correlation test for age, right‐down panel). [file CAI2-4-e70012-s004.tif]

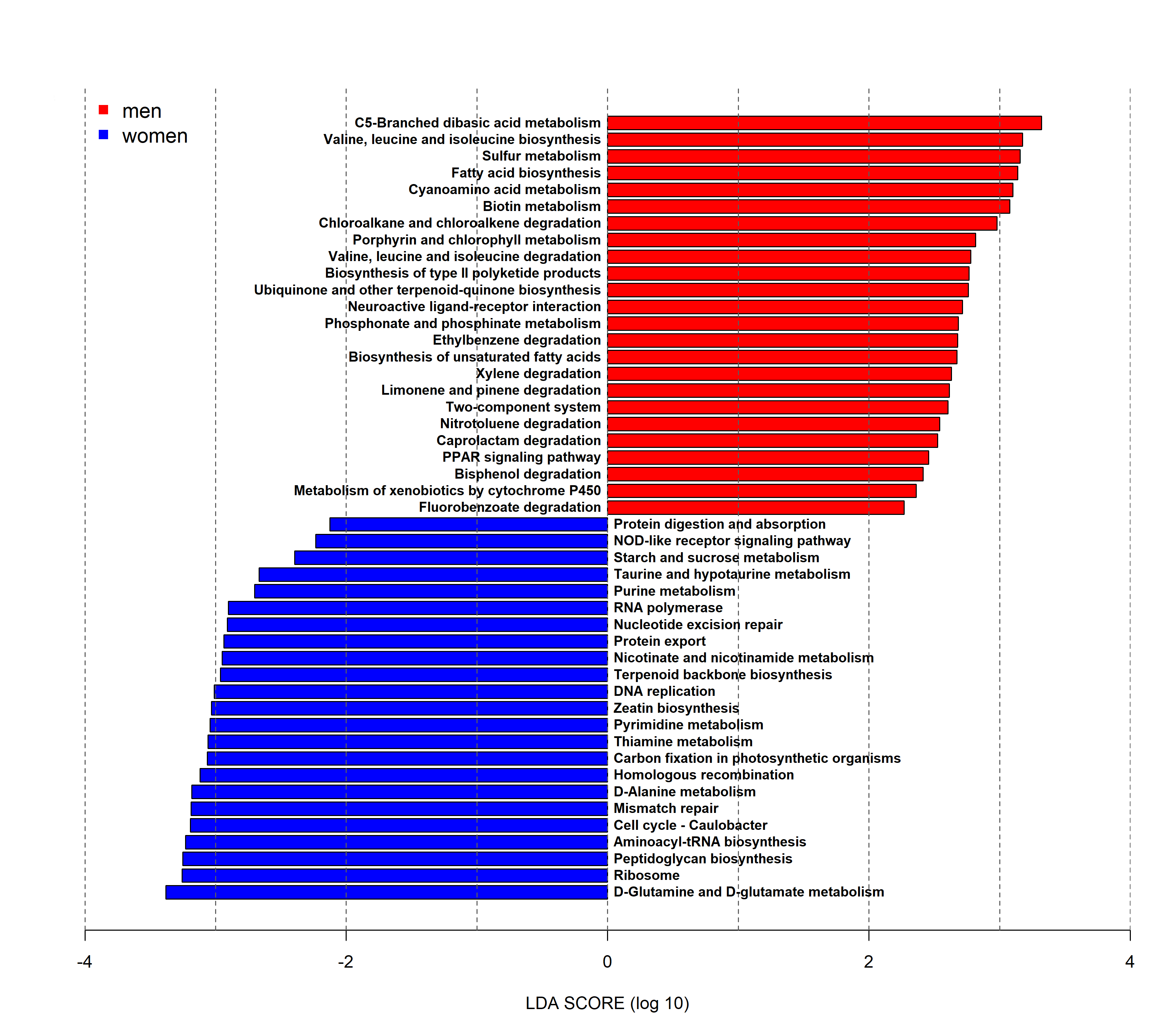

Supplement: Supplementary file 4 — Supporting Figure 4. Prediction functional analysis. KEGG pathways were significantly differentially enriched between healthy women (HW; n = 53, blue) and healthy men (HM; n = 41, red). [file CAI2-4-e70012-s003.tiff]
